# Supplementary material for: Reduction in Cold Stress in an Innovative Metabolic Cage Housing System Increases Animal Welfare in Laboratory Mice
Source: Animals (Basel). 2023 Sep 9;13(18):2866. doi: 10.3390/ani13182866 (PMC10525209; doi:10.3390/ani13182866)
Supplement: Supplementary file 1 [file animals-13-02866-s001.zip › animals-2540838-supplementary.pdf]

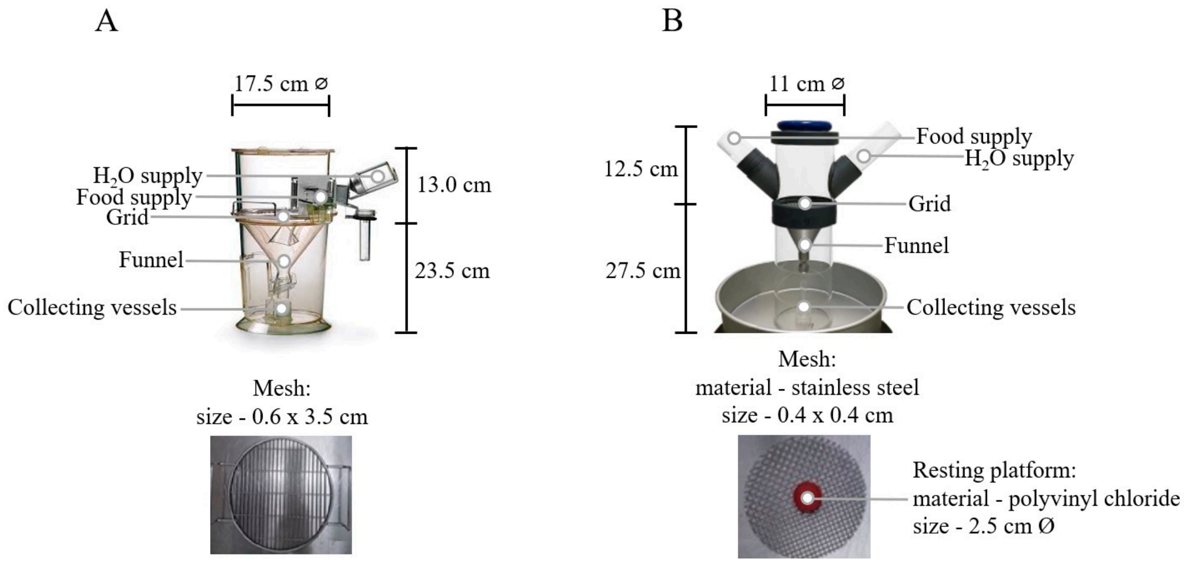

Figure S1. Comparison of applied metabolic cage types in the animal experiment.

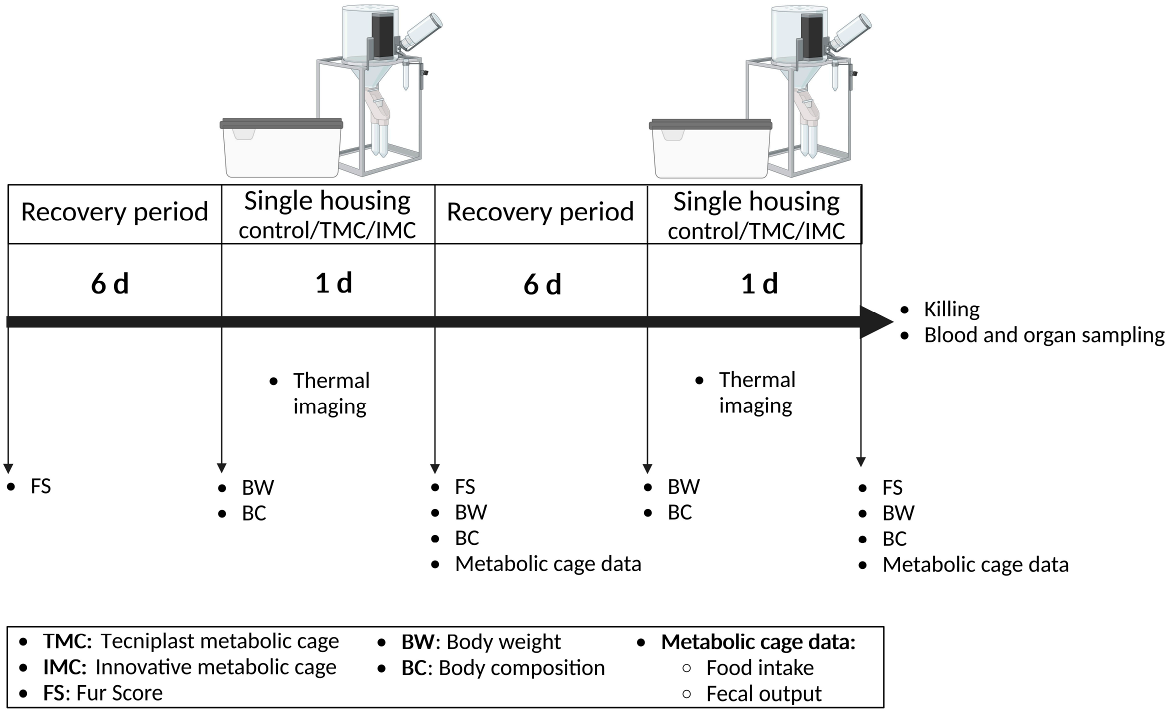

Figure S2. Study design of the conducted animal experiment shown as a time progression
